# Understanding Rice-*Magnaporthe Oryzae* Interaction in Resistant and Susceptible Cultivars of Rice under Panicle Blast Infection Using a Time-Course Transcriptome Analysis

**DOI:** 10.3390/genes12020301

**Published:** 2021-02-20

**Authors:** Vishesh Kumar, Priyanka Jain, Sureshkumar Venkadesan, Suhas Gorakh Karkute, Jyotika Bhati, Malik Zainul Abdin, Amitha Mithra Sevanthi, Dwijesh Chandra Mishra, Krishna Kumar Chaturvedi, Anil Rai, Tilak Raj Sharma, Amolkumar U. Solanke

**Affiliations:** 1 ICAR-National Institute for Plant Biotechnology, New Delhi 110012, India; visheshkumar08@gmail.com (V.K.); priybioinfo@gmail.com (P.J.); sureshkumarv1996@gmail.com (S.V.); suhaskarkute@gmail.com (S.G.K.); amithamithra.nrcpb@gmail.com (A.M.S.); trsharma1965@gmail.com (T.R.S.); 2 Jamia Hamdard, Hamdard Nagar, New Delhi 110012, India; mzabdin@jamiahamdard.ac.in; 3 ICAR-Indian Agricultural Statistics Research Institute, New Delhi 110012, India; singh.jyotika@gmail.com (J.B.); dwijesh.mishra@icar.gov.in (D.C.M.); kk.chaturvedi@icar.gov.in (K.K.C.); anil.rai@icar.gov.in (A.R.); 4 Indian Council of Agricultural Research, New Delhi 110012, India

**Keywords:** rice, panicle blast, RNA-Seq, *Magnaporthe*, cell wall modification, disease resistance

## Abstract

Rice blast is a global threat to food security with up to 50% yield losses. Panicle blast is a more severe form of rice blast and the response of rice plant to leaf and panicle blast is distinct in different genotypes. To understand the specific response of rice in panicle blast, transcriptome analysis of blast resistant cultivar Tetep, and susceptible cultivar HP2216 was carried out using RNA-Seq approach after 48, 72 and 96 h of infection with *Magnaporthe oryzae* along with mock inoculation. Transcriptome data analysis of infected panicle tissues revealed that 3553 genes differentially expressed in HP2216 and 2491 genes in Tetep, which must be the responsible factor behind the differential disease response. The defense responsive genes are involved mainly in defense pathways namely, hormonal regulation, synthesis of reactive oxygen species, secondary metabolites and cell wall modification. The common differentially expressed genes in both the cultivars were defense responsive transcription factors, NBS-LRR genes, kinases, pathogenesis related genes and peroxidases. In Tetep, cell wall strengthening pathway represented by PMR5, dirigent, tubulin, cell wall proteins, chitinases, and proteases was found to be specifically enriched. Additionally, many novel genes having DOMON, VWF, and PCaP1 domains which are specific to cell membrane were highly expressed only in Tetep post infection, suggesting their role in panicle blast resistance. Thus, our study shows that panicle blast resistance is a complex phenomenon contributed by early defense response through ROS production and detoxification, MAPK and LRR signaling, accumulation of antimicrobial compounds and secondary metabolites, and cell wall strengthening to prevent the entry and spread of the fungi. The present investigation provided valuable candidate genes that can unravel the mechanisms of panicle blast resistance and help in the rice blast breeding program.

## 1. Introduction

Rice is the staple food for more than two billion peoples worldwide. With a total production of nearly 755.473 million tons around the globe in 2019, China ranks first with 209.614 million tones followed by India with 177.645 million tons (Available online: http://fao.org/faostat (accessed on 22 December 2020)). Despite enormous production, it is estimated that 30% more rice is required by 2030 [1]. Worldwide 50% of the total yield loss is due to different environmental stresses. Amongst all, rice blast disease caused by a fungus *M. oryzae*, alone is responsible for 10% to 30% annual yield loss [2], which can reach up to 100% under favorable conditions by devastating the entire field of rice within 15 to 20 days after infection [3]. Rice blast symptoms appear on all parts of the plant including roots. In leaf blast, necrotic lesions are formed on the surface of the leaf, resulting in the reduction of photosynthetic rate, plant height, number of bearing panicles and also individual grain weight. Stem blast or panicle blast reduces the strength of the stem leading to barren panicles, broken necks and sterile grains. As it directly affects the panicles, it leads to complete yield losses and thus is a major threat to global rice production.

The most effective and environment-friendly approach for controlling blast disease is to use blast resistant cultivars of rice [4]. Traditional molecular breeding approaches have successfully developed blast resistant varieties [5]. However, the breakdown of resistance in these varieties require continuous efforts to identify novel genes or quantitative trait loci (QTLs) conferring broad spectrum resistance and also to understand the underlying mechanisms. Over 100 resistant (R) genes responsible for blast resistance have been identified and 25 of them have been cloned so far [6]. Two QTLs, panicle blast 6–1(qPb6–1) and panicle blast-bodao 1 (Pb-bd1) governing panicle blast resistance was identified earlier by molecular breeding approaches [7] and only one gene panicle blast 1 (Pb1) encoding an atypical CC domain containing nucleotide binding site leucine-rich repeats (CC-NBS-LRR) protein had been identified for panicle blast resistance [8]. Traditionally, map-based cloning using segregating populations like F_2_, recombinant inbred lines (RILs) and doubled haploid population was used for identification of blast resistant genes [9,10,11]. Along with it, new genomic technique like RNA sequencing (RNA-Seq) is proving to be useful and affordable to analyze differential expression patterns [12,13,14] and identification of resistant and susceptibility genes. Resistance against blast pathogen in rice is a combined effect of multiple regulons including transcription factors (myeloblastosis family of transcription factors (MYB), WRKY, ethylene response factor (ERF), etc.), signaling kinases, LRR genes, cell wall modification related genes, etc. [15,16,17]. Earlier, microarray analysis of blast resistant and susceptible lines also showed up-regulation of various defense response genes involved in resistance reactions in rice [18]. However, all these studies are related to leaf blast disease.

Though panicle blast is the most harmful type of blast disease, there are very few reports that are specifically targeting the panicle blast in rice. The disease response of leaf and panicle to blast infection is different and the varieties which are susceptible to leaf blast are resistant to neck blast and vice versa [19]. Some of the resistant cultivars at the seedling stage become susceptible to neck blast [20]. The well-known panicle blast resistant gene Pb1 governs “adult resistance” where rice plants are blast susceptible at a vegetative stage but are resistant at the reproductive stage and thus induces strong panicle blast resistance [8]. This warrants the identification of more such panicle blast resistant genes and related disease responsive pathways involved in rice. In the present study, panicle blast resistant and susceptible cultivars were studied by RNA-seq to compare global gene expression profiles with time-course analysis at three time points post inoculation of *M. oryzae*. It resulted in a plethora of differentially expressed loci involved in disease response. These genes primarily mapped to the major defense responsive pathways namely, pathogenesis, signal transduction, stress response, ROS production and detoxification, hormonal regulation, secondary metabolite synthesis, and cell wall modification. Besides these, some novel genes, functions of which are not yet characterized were also identified in the resistant cv. Tetep. The study finally proposed a novel complex transcript network responsible for resistance against the panicle blast disease.

## 2. Materials and Methods

### 2.1. Plant Materials, Fungal Material, Growth Conditions and Treatments

Seeds of indica rice cultivar Tetep (resistant to blast) and HP2216 (susceptible) were surface sterilized, germinated and allowed to grow in a greenhouse (25 ± 2 °C and 16 h light/8 h dark) till panicle formation. The disease resistant and susceptible phenotypes of these genotypes are well known and are characterized earlier in our laboratory. These plants were then used for inoculation of *M. oryzae* strain *Mo-ni-0025* (Dehradun, India). For inoculation, fungus culture was maintained on potato dextrose agar (PDA) media, for 10–12 days at 25 °C. Later, it was transferred to Mathur’s media and kept for 8 to 10 days at 25 °C for reproductive growth. The reproductive growth having conidia is then scrapped using 5 mL of autoclaved double distilled water and a conidial suspension (1 × 10^5^ conidia/mL) was prepared. Panicles at the neck were inoculated with the suspension by syringe inoculation technique which is standardized earlier in our laboratory and plants were kept in controlled conditions at the temperature (25 ± 2 °C) and 90% relative humidity in dark. Panicle samples of both Tetep and HP2216 cultivars were collected at 48, 72, and 96 h post infection (hpi) of blast pathogen along with mock inoculation using double distilled water. The time points for sample collection were identified by conducting experiments of disease infection, where the minor symptoms were visible on susceptible genotype at 48 hpi. Thus, 48 hpi was considered as early infection response to study time-course analysis. All samples were frozen in liquid nitrogen and stored at −80 °C till RNA isolation.

### 2.2. RNA Isolation and cDNA Preparation

All the inoculated panicle samples along with mock of both Tetep and HP2216 cultivars were used for total RNA isolation. Spectrum^TM^ Plant Total RNA Kit (Sigma-Aldrich, Co., St. Louis, MO, USA) was used for total RNA isolation according to the manufacturer’s protocol. The isolated RNA was analyzed for its quality by gel electrophoresis and quantified by spectrophotometer (Nano-Drop 2000, Thermo Fisher Scientific, Wilmington, DE, USA). RNA integrity number (RIN) was calculated by using Agilent 2100 Bioanalyzer (Agilent Technologies, Thermo Fisher Scientific Inc, Waltham, MA, USA) RNA samples with RIN greater than or equal to 7 were used for library and cDNA preparation. 1 µg of total RNA, isolated from the samples, was used to prepare cDNA using Applied Biosystems High-Capacity cDNA Reverse Transcription Kit (Thermo Fisher Scientific Baltics UAB, Vilnius, Lithuania) by following the manufacturer’s instructions.

### 2.3. Library Preparation, Illumina Sequencing and Processing of The Reads

The fragment library for RNA sequencing was prepared using Illumina True-Seq RNA Library Prep Kit (San Diego, California, USA) by following the given protocol. Each biological replicate was a pool of five independent panicles. Two biological replicates were used for each sample and thus 16 samples were sequenced. Illumina Hi-Seq 2000 platform was used to generate large amounts of sequencing data performing paired-end sequencing runs using 1 µg of good quality total RNA (RIN ≥ 7) to obtain 101 bp sequence length reads. Quality analysis of raw reads was performed using fast quality check (FastQC) (version 0.11.5) [21]. These raw reads were further processed by Trimmomatic (version 0.36) [22] for clipping adapter and low-quality sequences. Reads shorter than 36 bp were discarded and trailing bases below the quality 30 were trimmed. The reads were again subjected to quality check by FastQC before using for transcriptome analysis.

### 2.4. Reference Mapping, DEG and Pathway Analysis

Read mapping for gene expression analysis and identification of differentially expressed genes were performed using the Tuxedo pipeline. The Tuxedo pipeline includes TopHat2 [23], cufflinks, and cuffdiff. High quality trimmed reads were mapped to the rice reference genome (*Oryza sativa* L. ssp. japonica cv. Nipponbare) using TopHat2. Parameters during reference alignment of reads using TopHat2 were as follow: read mismatch as 2, read gap length as 2, read edit-distance as 2, minimum and maximum intron length as 50 and 500,000 respectively, maximum multi hits as 20, maximum insertion and deletion length of 3. Cufflink version 2.1.1 was used to estimate and assemble their abundance in RNA-seq samples using parameters like average fragment length of 200 bp, fragment length standard deviation of 80 bp and unlimited alignment was allowed per fragment (Available online: http://cole-trapnell-lab.github.io/cufflinks/cuffdiff/ (accessed on 22 June 2020)). In cufflink, RNA-seq reads were accepted and assembled to a parsimonious set called transcripts [24]. After mapping, reads were used to merge with their biological replicates using cuff-merge. Cuffmerge removes transcribed fragments that are artifacts in the experiment. Lastly, Cuffdiff version 2.1.1 was used to quantify transcripts in terms of fragments per kilobase of transcript per million mapped reads (FPKM) and to obtain a list of the significant differentially expressed loci.

Differentially expressed loci with fold change value of ≥ +2 and −2 and *p*-value of ≤ 0.01 were used to select the significant loci from the data. For further analysis, R-studio was used to generate a heatmap for the selected loci [25]. To compare between the two data sets and to identify novel, unique and common loci, Venn diagrams were prepared using InteractiVenn [26]. Pathway analysis for the significant differentially expressed loci (SDEL) was performed using the MapMan 3.5.1 with *O. sativa* L. ssp. *japonica* cv. Nipponbare MSU v7 mapping files [27] and Kyoto Encyclopedia of Genes and Genomes (KEGG) pathway. The chromosome map showing the locations of all the genes was generated using the Chromosome Map Tool available on Oryzabase (Available online: http://viewer.shigen.info/oryzavw/maptool/MapTool.do (accessed on 29 June 2020)).

### 2.5. Singular Enrichment Analysis and Gene Ontology Analysis

Singular enrichment analysis (SEA) and Gene ontology analysis was used to classify SDEL based on the molecular function, biological process, and the cellular component at different time points in both resistant and susceptible cultivars using AgriGo v2.0 [28]. AgriGo is an online tool and database used for Gene Ontology analysis, specially designed for agricultural studies.

### 2.6. Validation of Transcripts by Quantitative Real-Time PCR

Quantitative real-time PCR (qRT-PCR) was performed for validation of differentially expressed loci obtained from the RNA-seq data. Primers were designed according to the gene sequences present at the gene database using PrimerQuest (Integrated DNA Technologies, Coralville, IA, USA) tool (Supplementary Table S1). Light Cycler^®^ 480 II (Roche, Rotkreuz, Switzerland) was used to perform qRT-PCR with Brilliant III Ultra-Fast Sybr^®^ Green qPCR Master Mix from Agilent Technologies (Santa Clara, CA, USA). The reaction mixture of qRT-PCR was prepared using required amount of diluted cDNA as template, 0.3 µL of each primer, 15 µL 2 x SYBR Green Master Mix and 0.4 µL 6-Carboxy-X-Rhodamine (ROX) fluorescence dye (diluted as per the instructions given in manual) and nuclease-free water for making a total volume of 30 µL qRT-PCR reaction mix. This reaction mix was then used for qRT-PCR with the following thermal profile: 95 °C for 30 s, 60 °C for 15 s, and 72 °C for 20 s with 40 cycles of amplification. Three biological replicates were used for the experiment with three technical replicates of each biological replicate and the *18s* gene was used as a housekeeping gene for normalization. Relative fold change in the level of gene expression was calculated using the 2^−ΔΔCT^ method [29]. Significant variations between the mock and different time periods of infection were designated by the asterisk sign above the error bars, calculated by two-way ANOVA (*p*  <  0.05).

## 3. Results

### 3.1. Phenotype of Resistant and Susceptible Cultivars against M. oryzae

**Figure 1:**
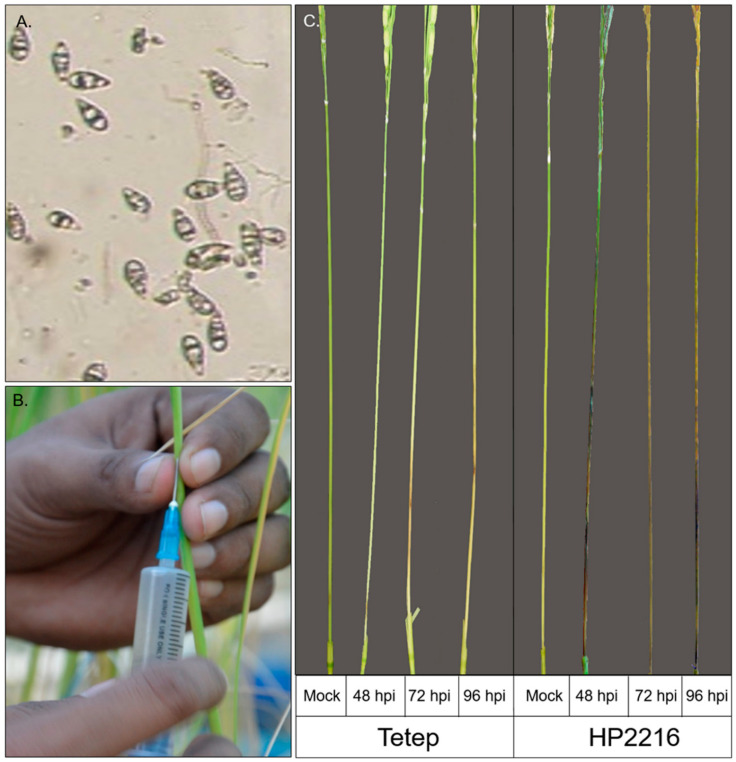
Inoculation of *M. oryzae* to rice panicles and disease phenotyping. (**A**) Suspension of *M. oryzae* spores, ready to infect the host. (**B**) Infection was given to the neck of rice panicle using 5 mL syringe. (**C**) Representative image of rice panicles showing the impact of pathogen on blast resistant (Tetep) and susceptible (HP2216) cultivars at different time intervals at 48 hpi, 72 hpi and 96 hpi (hpi= hour post infection) along with mock inoculated.

Flowering stage panicles of rice cultivars Tetep and HP2216, inoculated with *M. oryzae* strain *Mo-ni-0025* were harvested from the first node of the panicle from the top at different time points (Figure 1). Panicles infected with the suspension without any conidia showed wounding marks in both the cultivars, but no lesions were observed in either of them. In contrast, panicles infected with conidial suspension showed wounding marks in all the panicles as well as the disease reaction. While typical lesion formation was observed in cv. HP2216 48 hpi which further increased at 72 and 96 hpi, panicles from cv. Tetep were devoid of any lesion symptoms but showed just minor symptoms of hypersensitive response. The level of disease in panicle blast was scored using the method proposed by Ou [30] with 0–5 disease rating scale. Genotypes with scoring of lesions around 0 to 3 were considered as the resistant reactions and those with 4 to 5 were considered as susceptible reactions against the blast disease. Based on the scoring, Tetep was highly resistant with scores of mostly 0 and 1. In contrast, HP2216 panicles showed scores of mostly 4 and 5 confirming its susceptibility to *M. oryzae* infection (Supplementary Table S2).

### 3.2. Transcriptome Sequencing and Data Analysis

RNA-seq of the collected samples was performed using Illumina Hi-Seq 2000 platform. A standard protocol using a Tuxedo pipeline was used for removal of low-quality reads, alignment of reads along with the reference genome, and finding the differentially expressed loci. On an average 32 million raw reads were obtained from transcriptome sequencing run of each sample. After pre-processing (removal of adapters and low-quality reads) using Trimmomatic, 31 million 101 bp paired-end high quality reads per sample were obtained. This indicated a good quality of sequencing results. Approximately, 83% of total reads were aligned against reference genome *O. sativa* L. ssp. *japonica* cv. Nipponbare MSU v7 (Supplementary Table S3).

**Figure 2:**
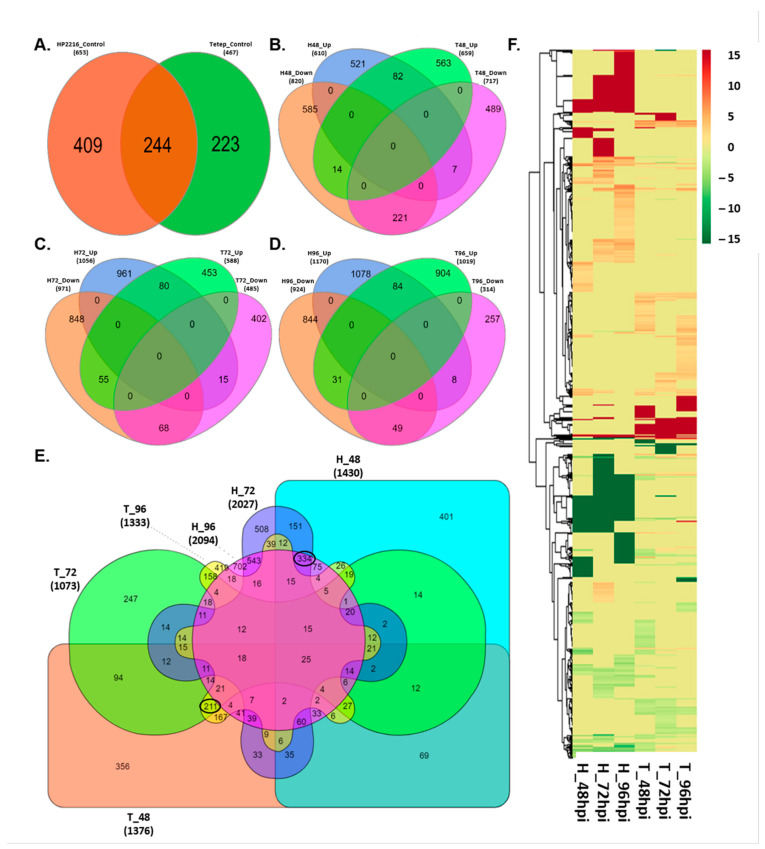
Differentially expressed genes in Tetep and HP2216. Study of plant-pathogen interaction using Venn and expression profiling in Tetep and HP2216 rice, infected with *M. oryzae*, at different time intervals. (**A**) Total expressed loci common and unique between Tetep and HP2216 in mock samples without infection (control). (**B**) Significant (FDR adjusted *p* ≤ 0.01) DELs (log2 fold change ≥2) common and unique in Tetep and HP2216 at 48 hpi. (**C**) Significant DELs common and unique in Tetep and HP2216 at 72 hpi. (**D**) Significant DELs common and unique in Tetep and HP2216 at 96 hpi. (**E**) Significant DELs common and unique between Tetep and HP2216 at all three-time intervals. (**F**) Heat map of significant (FDR adjusted *p* ≤ 0.05 & log2 fold change ≥2) DELs of Tetep and their respective log2 fold change in HP2216. Red represents up-regulated loci and green represents down-regulated loci.

Number of significant differentially expressed loci in HP2216 were 1430, 2027 and 2094 and in Tetep were 1376, 1073 and, 1333 loci at 48, 72 and 92 hpi, respectively in comparison with mock (Figure 2, Supplementary Table S4). These datasets were then merged to form a table with the pair-wise comparison which showed a total of 5205 common differentially expressed loci in both genotypes. The distribution of SDEL over 12 chromosomes in rice was observed on each chromosome for both resistant and susceptible genotypes (Supplementary Figures S1 and S2).

### 3.3. Differentially Expressed Loci among Blast Resistant and Susceptible Rice Cultivars

The number of up-regulated and down-regulated loci varied across the different time points in both HP2216 and Tetep. Most of the loci showed similar expression in control tissues of both cultivars, as both are indica rice. A total of 244 SDEL were common to both the cultivars while 409 and 223 loci were uniquely expressed in the HP2216 and Tetep, respectively (Figure 2A). However, across different hpi, the number of common down-regulated loci were decreasing (i.e., 221, 69, and 48 differentially expressed loci (DELs) respectively at 48, 72 and 96 hpi) while the number of common up-regulated DELs were similar (i.e., 82, 80 and 84 DELs at 48, 72 and 96 hpi) between the resistant and susceptible cultivars (Figure 2B–D). This data indicated that the plant-pathogen interaction may trigger the genes responsible for the response of the plant to infection in their early stage of interaction, and after reaching their threshold value, the expression of genes again restores to their normal form. When we compared the loci from all the three time points of HP2216 and Tetep together in a single Venn diagram, a broad visualization of loci distributed among all the time points could be observed. By comparing the loci from all the three time points, it was found that a total of 211 loci were specific to Tetep and a total of 334 loci were specific to HP2216 (Figure 2E). This preliminarily suggests that there are specific genes in both the cultivars that could possibly play a role in plant pathogen interaction which could lead to resistance in Tetep and susceptibility in HP2216. 

Tetep cultivar responded to blast pathogen by up-regulation of 659 loci at 48 hpi which got reduced to 588 at 72 hpi and rose to 1019 at 96 hpi. However, the number of down-regulated loci was reduced from 717 at 48 hpi to 485 at 72 hpi and 314 at 96 hpi. In contrast, there was a continuous increase in the number of both up-regulated and down-regulated loci in HP2216 as the disease infection progressed. The up-regulated loci were 610 at 48 hpi, 1056 at 72 hpi and 1170 at 96 hpi; whereas down-regulated loci were 820 at 48 hpi which then increased to 971 at 72 hpi and 924 at 96 hpi. This data supports that in Tetep, some loci show early up-regulation after infection (Figure 2B–D).

### 3.4. Gene Ontology Analysis

**Figure 3:**
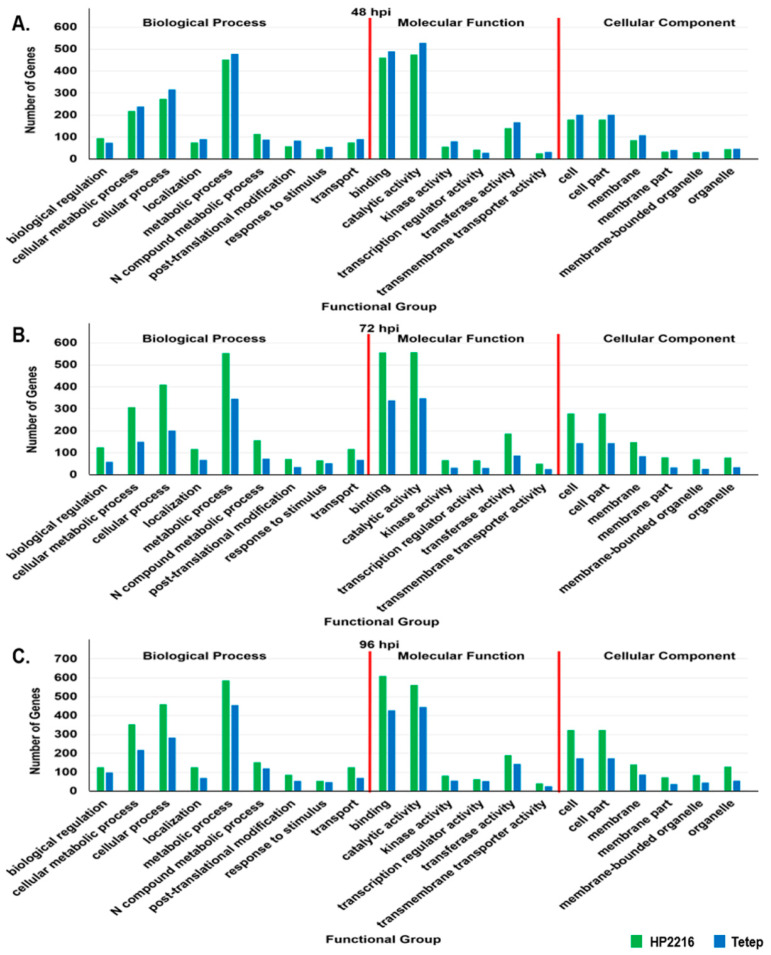
Gene Ontology for significant differentially expressed genes. Graphical representation of significant differentially expressed loci (SDEL; FDR adjusted *p* ≤ 0.01) in HP2216 comparative to Tetep present in different biological processes, molecular functions and cellular components of all significant GO terms at all three-time intervals. (**A**) Significant GO terms in HP2216 comparative to Tetep at 48 hpi. (**B**) Significant GO terms in HP2216 as comparative to Tetep at 72 hpi. (**C**) Significant GO terms in HP2216 comparative to Tetep at 96 hpi. GO terms for biological processes are biological regulation (GO:0065007), cellular metabolic process (GO:0044237), cellular process (GO:0009987), localization (GO:0051179), metabolic process (GO:0008152), nitrogen compound metabolic process (GO:0006807), post-translational modification (GO:0043687), response to stimulus (GO:0050896) and transport (GO:0006810). GO terms of molecular function are binding (GO:0005488), catalytic activity (GO:0003824), kinase activity (GO:0016301), transcription regulator activity (GO:0030528), transferase activity (GO:0016740) and transmembrane transporter activity (GO:0022857). GO terms of cellular components are cell (GO:0005623), cell part (GO:0044464), membrane (GO:0016020), membrane part (GO:0044425), membrane bound organelle (GO:0043227) and organelle (GO:0043226). The green bars represent for HP2216 and blue bars represent for Tetep.

Gene ontology (GO) enrichment analysis was conducted to identify the biological functions of the SDEL of Tetep and HP2216 cultivars. The loci were categorized into 3 major categories: biological process (BP), molecular function (MF), and cellular components (CC); and further subcategories of each main category (Supplementary Figure S3A,B). Overall, *M. oryzae* infection in rice caused an expression of a higher number of genes (SDEL) involved in various processes in HP2216 than in Tetep. Both cultivars showed similar patterns where most of the loci were present in binding and catalytic activity under MF, metabolic process and cellular process under BP, and cell part in CC. Further, to understand the real-time molecular response towards disease infection which results in different responses of resistant and susceptible cultivars, the analysis of these loci at different time intervals was carried out and the same has been depicted in Figure 3.

Comparative results of each time point in both cultivars for all three gene ontology categories were shown in Supplementary Figure S4. In Tetep, there was a higher number of SDEL at 48 hpi than HP2216 which gradually declined at 72 hpi and again restored at 96 hpi. However, the number of SDEL in categories like, response to stimulus and transmembrane transporter activity was decreased at both 72 hpi and 96 hpi. In Tetep, the exceptional category was of transcription regulator activity where the number of SDEL increased as the duration after infection increased. The number of genes (SDEL) in BP at 48, 72 and 96 hpi in HP2216 showed a gradual increase except for the loci in response to stimulus which declined after 72 hpi. A similar pattern was observed for the number of SDEL in MF and CC categories in HP2216, except for transmembrane transporter activity which declined after 72 hpi. 

### 3.5. Validation of Transcripts Using Quantitative Real-Time PCR 

**Figure 4:**
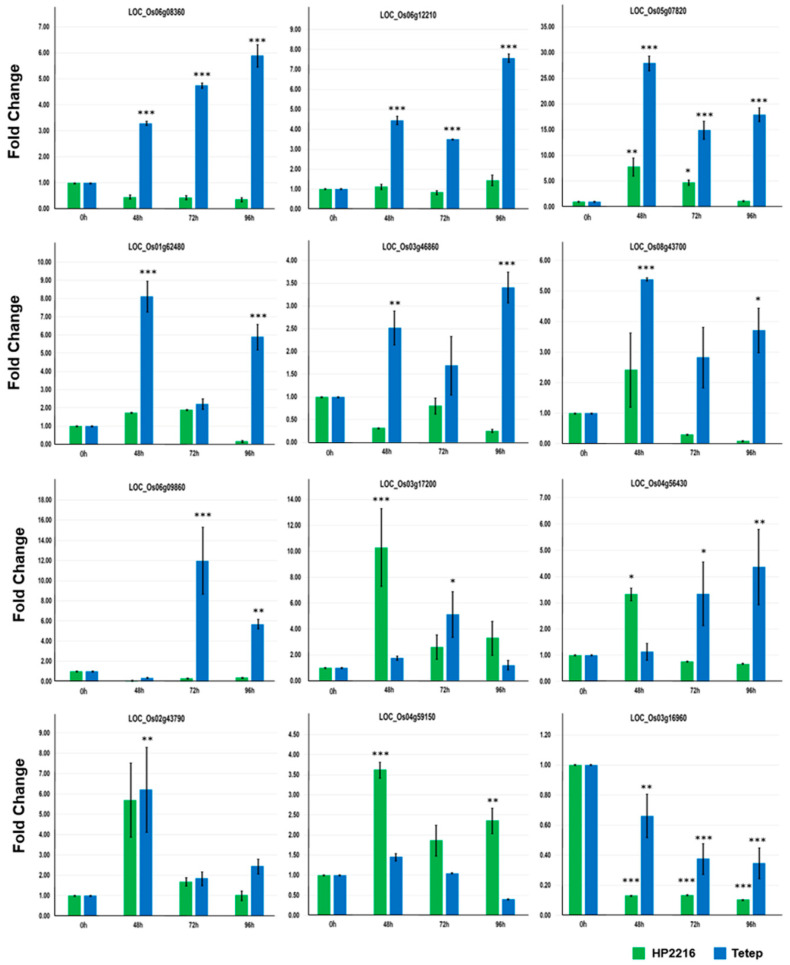
qRT-PCR validation of selected differentially expressed genes. qRT-PCR validation of significant differentially expressed loci (SDEL) in HP2216 and Tetep upon *M. oryzae* infection. The green and blue bars represent the absolute fold change of HP2216 and Tetep, respectively. *18s* was used for transcript normalization. Standard error bar shows the standard deviation for three replicate assays. LOC_Os06g08360 (ethylene-responsive element-binding protein), LOC_Os06g12210 (helix-loop-helix DNA-binding domain containing protein), LOC_Os05g07820 (leucine-rich repeat receptor protein kinase EXS precursor), LOC_Os01g62480 (laccase precursor protein), LOC_Os03g46860 (helix-loop-helix DNA-binding protein), LOC_Os08g43700 (OsSAUR36 –small Auxin-responsive SAUR gene family member), LOC_Os06g09860 (expressed protein), LOC_Os03g17200 (plant-specific domain TIGR01589 family protein), LOC_Os04g56430 (cysteine-rich receptor-like protein kinase), LOC_Os02g43790 (ethylene-responsive transcription factor), LOC_Os04g59150 (peroxidase precursor), LOC_Os03g16960 (cysteine-rich repeat secretory protein 55 precursor).

The transcriptome data obtained through RNA-Seq was validated by qRT-PCR analysis. We selected 12 genes which included both up-regulated and down-regulated based on biological functions and variations in their expression patterns. Some of the important genes selected for validation were LRR receptor protein kinase precursor, laccase precursor, peroxidase precursor, ethylene responsive transcription factor, ethylene responsive element binding protein and cysteine-rich receptor-like protein kinase which were known to be disease responsive. Expression of all these genes by qRT-PCR analysis showed good correlation with RNA-Seq data (Figure 4). For example, LRR receptor protein kinase precursor (LOC_Os05g07820) showed more than 25-, 15- and 17-fold up-regulation at 48, 72 and 96 hpi, respectively in Tetep whereas in HP2216 the up-regulation was far less. Similarly, another gene cysteine-rich repeat secretory protein 55 precursor (LOC_Os03g16960) which showed down-regulation in transcriptome data in both Tetep and HP2216 also showed down-regulation in qRT-PCR analysis.

### 3.6. Pathway Analysis 

For comparative analysis of defense mechanisms in rice blast resistance (Tetep) and susceptible (HP2216) cultivars, pathway analysis was carried out. The important pathways where differentially regulated loci involved were defense response, cell wall modification, hormonal response, transcription regulation, LRR signaling and secondary metabolite synthesis which are described here in detail.

#### 3.6.1. Primary Defense Response through Reactive Oxygen Species

**Figure 5:**
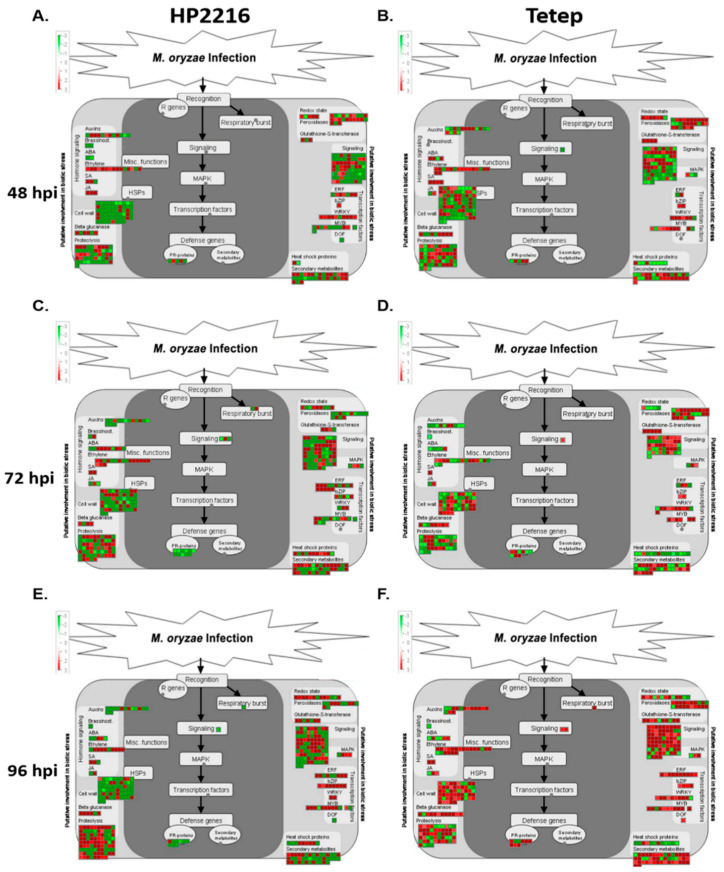
MapMan analysis for significant differentially expressed genes. MapMan overview of significant (FDR adjusted *p* ≤ 0.05 & log2 fold change ≥2) differentially expressed loci (SDEL) involved in biotic stress pathway, in HP2216 and Tetep, at different time intervals of *M. oryzae* infection. SDEL are binned to MapMan functional categories and values represented as log2 fold change values. (**A**,**B**) MapMan overview of SDEL in biotic stress pathways in HP2216 and Tetep respectively at 48 hpi. (**C**,**D**) MapMan overview of SDEL in biotic stress pathways in HP2216 and Tetep respectively at 72 hpi. (**E**,**F**) MapMan overview of SDEL in biotic stress pathways in HP2216 and Tetep respectively at 96 hpi. Red color represents up-regulated loci and green represents down-regulated loci.ABA, abscisic acid; JA, jasmonic acid; SA, salicylic acid; HSPs, heat shock proteins; R genes, resistance genes; ERF, ethylene response factor; bZIP, basic region-leucine zipper; WRKY, WRKY family of transcription factors; MYB, myeloblastosis family of transcription factors; DOF, DNA-binding with one finger plant specific transcription factors; MAPK, mitogen-activated protein kinase; PR-protein, pathogenesis-related protein.

During infection of blast pathogen on resistant and susceptible cultivars, primary response leads to respiratory burst that includes redox state genes like glutathione, glutaredoxin, thioredoxin, redoxin glutathione S-transferases and peroxidase (Figure 5). In both, Tetep and HP2216 cultivars, the number of SDEL related to primary response was gradually increased post infection. However, the number of up-regulated genes was more in Tetep. One of the most important genes identified for stress response in the study was stress responsive A/B Barrel domain containing protein (LOC_Os11g05290), which showed more than 4-fold up-regulation in Tetep but no expression in HP2216. In Tetep, the number of up-regulated loci related to redox state was 6 at 48 hpi, 7 at 72 hpi and 8 at 96 hpi, whereas this number was comparatively less in HP2216 at initial response as 4 at 48 hpi, 5 at 72 hpi but increased at later stage as 10 at 96 hpi. These SDEL includes up-regulated loci like cytochrome b5-like Heme/Steroid binding domain containing protein involved in monooxygenase cycle [31], glutaredoxin family proteins involved in protection against photooxidative stress [32], glutathione reductase which provides resistance against oxidative stress [33], OsGrx_S2-glutaredoxin subgroup III, SOUL heme-binding protein that plays a key role in stress signaling and plants primary metabolic pathway [34], tetratricopeptide repeat thioredoxin like proteins, TTL1 and TTL3 involved in osmotic stress tolerance in plants [35], L-ascorbate oxidase precursor, protein disulfide isomerases, monodehydroascorbate reductase which helps in reactive oxygen species (ROS) detoxification and play key role in maintaining oxidative stress in plants [36], electron carrier/ protein disulfide oxidoreductase, etc. At the same time infected plants exhibited down-regulation of several genes such as glutathione synthetase working in nitrogen metabolism [37], cytochrome b561, glutathione S-transferase, glutaredoxin family protein, peroxiredoxin type 2 to control the oxidative stress, L-ascorbate peroxidase, catalase domain containing protein, thioredoxin, etc. However, the number of differentially expressed loci related to respiratory burst was more in resistant cultivar compared to susceptible one (Figure 5A–F). Number of up-regulated SDEL after the infection was higher in Tetep than HP2216 that goes on increase as the disease progresses. Most importantly, NADPH dependent oxidoreductase which plays a role in detoxification showed near about 5-fold up-regulation at 48 hpi which increased to more than 6.6 fold at 72 hpi in Tetep but its expression was not detected in HP2216 at 48 hpi and showed up-regulation only at 72 and 96 hpi. This indicates a strong response of resistant cultivar Tetep to activate respiratory burst related genes against panicle blast infection.

#### 3.6.2. Transcript Level Changes in Cell Wall Proteins

ROS signaling plays a key role in inducing cell wall modification mediated defense. Our results indicated many genes related to cell wall biogenesis and modifications were up-regulated in Tetep as compared to HP2216. These cell wall related SDEL include cell wall proteins, cellulose synthesis (cellulose synthase), cell wall degradation (cellulases, β -1,4-glucanases, mannan-xylose-arabinose-fucose, pectate lyases, and polygalacturonases), cell wall hemicellulose synthesis, cell wall pectin esterase, and cell wall precursor synthesis (Supplementary Table S4). Transcriptional level changes of cell wall modification transcripts were one of the highest sets of SDEL that showed a change in expression upon infection. In resistant cultivar, major cell wall related SDEL showed up-regulation while opposite trend was observed in susceptible cultivar HP2216 at 48, 72 and 96 hpi. For instance, in Tetep, several genes of glycosyl hydrolase family, glycosyltransferase family, Powdery Mildew Resistance 5 (PMR5), dirigent, tubulin, cellulose synthase and other cell wall proteins were highly induced immediately after infection but the number of genes, as well as their level of induction was less in HP2216. Two glycosyltransferase 8 domain containing protein coding genes (LOC_Os07g48830 and LOC_Os07g45260) involved in cell wall thickening were up-regulated in Tetep at 72 hpi and continued to express even at 96 hpi. The expression of these genes was negligible in HP2216. In addition, Tetep showed up-regulation of many MYB family transcription factors (LOC_Os06g02250, LOC_Os05g49310, LOC_Os05g37050, LOC_Os05g50350, LOC_Os01g19330) working in regulation of secondary cell wall biogenesis, but their expression in HP2216 was low. In Tetep, as many as six chitinase family protein precursors were up-regulated at both 48 and 72 hpi with an increased expression level at 72 hpi. In contrast, the number of chitinase family protein precursor genes in HP2216 were high at the initial stage of infection but its expression was reduced at later stages. Cell wall degradation loci were composed of both up and down-regulated SDEL at all the time points in both the cultivars. In resistant cultivar, four SDEL involved in cell wall precursor pathway showed early (48 hpi) up-regulation compared to susceptible cultivar. Cell wall modification related SDEL at 48 and 72 hpi consist of both up and down-regulated transcripts but at 96 hpi majority of SDEL were up-regulated in resistant cultivar while down-regulated in susceptible one. 

#### 3.6.3. Signal Transduction

Plant signaling plays a crucial role in providing resistance against pathogenesis in plants. This helps in activating and maintaining the innate immunity of the plant and involves receptor-like kinases (MAPKs), and leucine rich repeats (LRR) signaling. Figure 5 showing the number of SDEL involved in MAPK pathways and LRR signaling depicts the gradual increase of these SDEL from 48 hpi to 96 hpi in both Tetep and HP2216 cultivars. Signaling molecules like receptor kinase, G proteins, calcium binding protein, phosphoinositides and MAPK showed a constant increase in expression level from 48 hpi to 96 hpi in Tetep. The most important signaling protein of disease response brassinosteroid insensitive 1-associated receptor kinase 1 (BAK1) precursor (LOC_Os11g31540) and leucine-rich repeat transmembrane protein kinase (LOC_Os05g07820) playing crucial role in defense against various stresses were expressed both in Tetep and HP2216 at all the three time points studied here. Mitogen-activated protein kinase works as a signaling molecule for the transduction of extra cellular stimulation of cell into intra-cellular response that leads to activation of transcription factors in plants against any pathogenic invasion (Supplementary Figure S5). Tetep showed significant up-regulation of 13 SDEL of LRR genes at 48 hpi, 12 SDEL related to LRR genes at 72 hpi and 13 SDEL at 96 hpi with increased level of expression as the disease progresses. It has been reported in previous studies that the LRR gene family has the most predominant R-genes, responsible for providing resistance against many pathogens in plants. The resistant cultivar Tetep has more than 455 NBS-LRR genes that are largely responsible for its blast resistance [38].

#### 3.6.4. Regulation by Transcription Factors

Several transcription factors like ERF, MYB and WRKY activates the defense responsive genes after perceiving the signals from defense signaling pathways in plants. Importantly, ERF transcription factors are responsible for the regulation of pathogenesis related (PR) genes and integrate the signals received from ethylene (ET) and jasmonic acid (JA). Infection of *M. oryzae* in Tetep and HP2216 induced a large number of ERF, MYB, and WRKY TFs at all the stages of infection with variation in their numbers. *M. oryzae* infection in Tetep caused up-regulation of large number of Basic Region-Leucine Zipper (bZIP), WRKY and MYB transcription factors at 48 hpi (Figure 5B) and the expression of these genes increased at 72 hpi and 96 hpi (Figure 5D,F). The number of up-regulated ERF TFs increased to a great extent at 96 hpi. MYB TFs regulate the development, differentiation and metabolism of plant cells and also play a vital role in stress response [39]. It activates the genes responsible for immune signaling, phenylpropanoid biosynthesis, and ROS production due to biotic stress for defense against the pathogens in plants [40,41]. WRKY genes which are well known for their role in defense response against *M. oryzae* and *Xanthomonas oryzae* were also predominantly up-regulated in Tetep. For example, WRKY71, WRKY118, WRKY55, WRKY1, WRKY83 and WRKY7 were highly induced in Tetep after infection. At the same time there was down-regulation of WRKY43 and WRKY35 in Tetep. In HP2216, early stage of infection was characterized by down-regulation of more MYB TFs and up-regulation of more WRKY TFs whereas the large number of ERF TFs was up-regulated at 72 hpi (Figure 5A,C). At 96 hpi also, more MYB TFs continued to show up-regulation (Figure 5E). In addition, observation of down-regulation of some of the TFs in both genotypes indicates that they might play a negative role in disease response as well. 

#### 3.6.5. Hormonal Regulation

JA, SA, and ET are important hormonal signal molecules for biotic stress response during plant pathogen interactions [42]. *M. oryzae* infection to both resistant and susceptible rice cultivars showed major changes in the expression of SDEL involved in ET metabolism at early and late infection stages. At an early stage of infection (48 hpi), several genes involved in the biosynthesis of methyl salicylate, jasmonate, abscisic acid (ABA) and ET were up-regulated in Tetep (Figure 5B). Genes for JA and SA synthesis continued to express at high level at all the stages, but there was slight down-regulation of genes for ET synthesis at 72 hpi which got up-regulated again at 96 hpi (Figure 5D,F). In comparison, the number and level of expression of genes involved in JA, SA and ET was less in HP2216 at all the stages of infection (Figure 5A—C). Genes encoding for flavonol synthase/flavanone 3-hydroxylase involved in phenolic defense against fungi and insects [43] were up-regulated and gibberellin 3-β-dioxygenase regulating the level of gibberellins was down-regulated in Tetep. Overall, the number of expressed genes for SA, JA and ET was higher at the early stage of infection which declined at 72 hpi but again increased at 96 hpi showing complex regulation of hormones in Tetep cultivar. In HP2216, the genes for S-adenosyl-L-methionine: benzoic acid carboxyl methyltransferase involved in the biosynthesis of methyl salicylate continued to express at 72 hpi and 96 hpi as well, however, genes involved in JA were not up-regulated during the latter course of infection (Figure 5C,E). ET synthesis also decreased in HP2216 after 72 hpi as evident by reduced expression of its biosynthetic genes at 96 hpi.

#### 3.6.6. Activation of Secondary Metabolites

Secondary metabolites in plants such as flavonoids, terpens, lignins, and phenylpropanoids are the products of primary metabolic pathways that are necessary for defense against pathogen infection [44]. Disease response in Tetep was characterized by induction of genes that are involved in the biosynthesis of some of the major metabolites like chalcone, anthocyanin, isoflavonoid phytoalexin, terpenes, lignins, etc. and down-regulation of genes related to anthocyanidin 3-O-glucosyltransferase, dihydroflavonol-4-reductase and cycloartenol (Supplementary Figure S6B). In subsequent stages of infection at 72 and 96 hpi (Supplementary Figure S6D,F), the number of SDEL for synthesis of secondary metabolites (e.g., LOC_Os04g27430, LOC_Os03g24690, LOC_Os04g42830, LOC_Os08g04540, LOC_Os10g20610, LOC_Os11g16260, LOC_Os11g42480) and also number of secondary metabolites increased which could be responsible for resistance against panicle blast disease. In an exception, expression of phenylalanine ammonia-lyase (LOC_Os02g41670) involved in lignin synthesis was higher at 48 hpi but decreased to zero at succeeding stages of infection. Comparatively, the initial response of HP2216 at 48 hpi was similar to Tetep (Supplementary Figure S6A). However, the number of genes involved in all these pathways increased at 72 hpi and again decreased at 96 hpi (Supplementary Figure S6C,E). Moreover, flavonoids and phenylpropanoids biosynthesis related genes increased in number as well as expression level during infection in Tetep while decreased in HP2216, suggesting that it could be one of the mechanisms of resistance in Tetep.

### 3.7. Expression of Novel Genes Specific to Resistant Cultivar Tetep

There are large number of genes in Tetep along with NBS-LRR genes responsible for blast disease resistance but are not yet characterized. We, therefore, screened the transcriptome data to identify genes which were up-regulated significantly in Tetep post infection but were either negatively regulated or not expressed at all in HP2216. Thus, comparative analysis of transcriptome data helped to identify several novel genes like von Willebrand factor type A (VWA) domain containing genes (4 fold up-regulation in Tetep), eukaryotic aspartyl protease domain containing gene (5 fold up-regulation in Tetep), auxin-induced in root cultures protein 12 (AIR12) gene that codes for a dopamine β-monooxygenase N-terminal (DOMON) domain containing protein (3 fold up-regulation in Tetep), Armadillo/β-catenin repeat family protein (4 fold up-regulation in Tetep) etc. whose function in disease response is not yet well characterized. Characterization of these genes may provide in-depth insight into panicle blast resistance. 

## 4. Discussion

Considering the devastating nature of rice blast disease, it is necessary to develop rice cultivars resistant to blast in order to ensure global food security. The foremost thing to develop such resistant varieties is to identify the genes responsible for resistance and also to understand the resistance mechanisms. Researchers have explored large number of resistant genotypes and have identified more than 100 R genes providing resistance against *M. oryzae* infection [1] and many R genes and defense response genes have been identified in the rice genome [45,46]. Among the widely used sources of resistance, Tetep is the most important rice genotype as it provides durable and broad-spectrum resistance against rice blast [38,47]. The blast resistance gene *Pi54* has already been cloned and characterized from this genotype using a map-based cloning approach [9]. Recently, as many as 455 NBS-LRR genes have been identified in Tetep and 219 of them have been cloned and characterized for resistance against 12 different strains of *M. oryzae* [38]. This report re-established the Tetep as a reliable source of resistance for utilizing in breeding programs. Although resistance in Tetep or any other resistant line is largely attributed to R genes, the upstream or downstream pathways of the disease response and the actual mechanisms of resistance are yet to be characterized. Here, to understand the blast resistance mechanisms and genes responsible, a comparative panicle blast transcriptome analysis of Tetep along with a susceptible cultivar HP2216 at different time points after the infection was carried out. In order to understand the early and late response of plants through expression of disease responsive genes, 3 different stages of infection i.e., 48, 72 and 96 hpi and mock inoculation as control were targeted for expression analysis. The present study revealed a number of genes involved in various disease responsive pathways which might be responsible for the differential nature of Tetep and HP2216 during panicle blast disease. Infection of *M. oryzae* in HP2216 led to the formation of lesions at all the wounding sites. The size of lesions increased during disease progression indicating that the susceptible plants could not arrest the growth and spread of fungi to neighboring tissues (Figure 1). This ultimately resulted in reduced photosynthetically active area at the neck and also movement of nutrients to the panicle that has direct impact on the yield. On the other hand, the resistance of Tetep at the phenotypic level is characterized by development of hypersensitive response, a form of programmed cell death, at the site of infection and thus, restricts the spread of fungi to healthy tissues [48]. This phenotypic resistance is the ultimate result of several molecular, biochemical and physiological responses involving various pathways and accumulation of reactive oxygen species, antimicrobial compounds and phytoalexins at the infection site [49].

Comparative transcriptome analysis of Tetep and HP2216 cultivars after infection revealed that a total 2491 genes were differentially expressed in Tetep but the number of such genes was 3553 in HP2216 which was significantly higher than Tetep. This supports the previous reports showing that the infection has a greater impact on the global gene expression profile of both susceptible and resistant cultivars, but the magnitude of the impact is higher in susceptible rice cultivar [50]. In susceptible plants, comparatively more energy is diverted to stress management by defense response which is one of the reasons that eventually affect the growth and yield of crops [51]. In contrast, less effect on the global transcriptome of Tetep due to infection explains its effective stress management and low growth penalty. Moreover, most of the differentially expressed genes in Tetep were involved in one or the other pathways of disease response. The higher number of differentially expressed genes related to ethylene hormone metabolism in resistant cultivar upon infection of *Magnaporthe* in panicle is highly similar to leaf blast infection as reported earlier in resistant near isogenic lines (NIL) [16]. The highly up-regulated genes during the early stage of infection in Tetep included transcription factors like WRKY, MYB and ERF; defense genes like chitinases, laccases, lipoxygenases, peroxidases, phenyl alanine ammonia lyase, and pathogenesis related (PR) genes; MAP kinases, genes involved in respiratory burst and signaling, etc. MAP kinases and WRKY transcription factors are involved in signal transduction to activate defense gene expression [49]. MYB transcription factors act as positive regulators of lignin biosynthesis that helps to strengthen the cell wall. Immediate up-regulation of all these genes at 48 hpi shows a quick response of Tetep to infection as compared to HP2216. Expression of all these genes was either stable or decreased in later stages of infection in Tetep, however, HP2216 continued their up-regulation in increasing order throughout the disease progression. This indicates termination of resource allocations in the resistant line to defense response at an early stage of infection to avoid additional burden on plant machinery. Re-programming of plant metabolic processes to suppress the defense response has been earlier reported in rice against *Magnaporthe* infection which helps fungi to colonize plant tissue [52]. In resistant cultivar Tetep, there was up-regulation of genes involved in the synthesis of secondary metabolites like flavonoids, terpenes, and phenylpropanoids for synthesis of phytoalexins, lignin, and cutin which are responsible for antimicrobial activity of plant cells and also strengthening physical barrier. It has been already reported that biosynthesis of secondary metabolites such as phenols, phenylpropanoids and lignin imparts resistance against fungal diseases in rice [49]. 

Several reports in various crops have suggested cell wall modification and strengthening by deposition of lignins, callose, peroxidases, phenols and other chemically modified cell wall materials as one of the major strategies of disease resistance [49,53,54]. Our results corroborate the similar mechanism of resistance in Tetep against *M. oryzae*. For example, Tetep cultivar showed significant up-regulation of genes like L-ascorbate peroxidase (2.3 fold), thylakoid-bound ascorbate peroxidase (OsAPx3) (2.6 fold), monodehydroascorbate reductase (2.9 fold), and many peroxidase precursors involved in the synthesis of peroxidases and detoxification of ROS at all the stages of infection, however, in HP2216 these genes down-regulated at later stages showing suppression of their synthesis by pathogen. 

**Figure 6:**
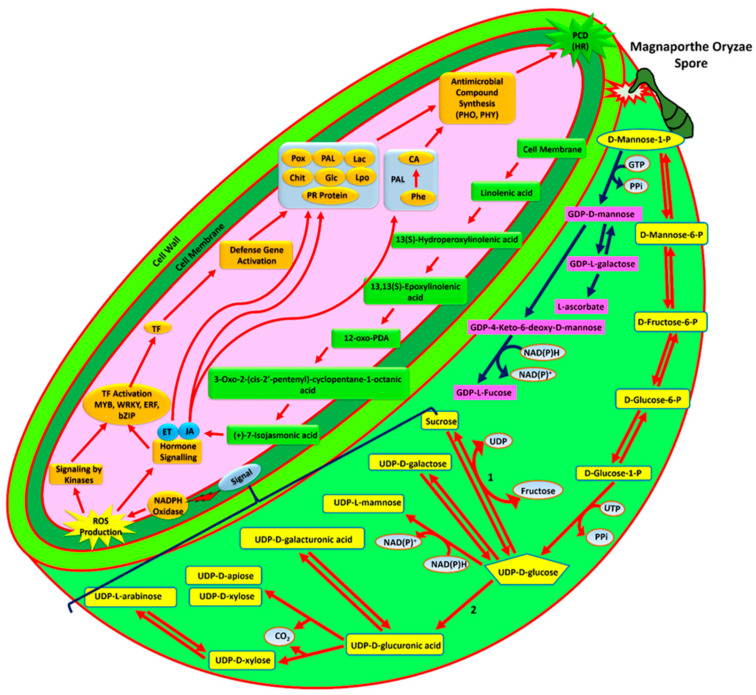
Overview of mechanism of rice blast resistance in Tetep at cellular level. A model to represent the resistance against *M. oryzae* infection in resistant rice cultivar Tetep. *M. oryzae* spore in the form of conidia attaches to the surface of tissue using a hook like structure and grows a germ tube to penetrate inside the epithelial cell layer. This stimulus triggers a cell wall precursor synthesis pathway leading to generation of two NADPH molecules and their subsequent oxidation for ROS production. This activates the kinase and hormone signaling molecules. Simultaneously, jasmonic acid production starts at the cell membrane of infected cells that triggers downstream hormone signaling. Kinase signaling molecules activate transcription factors (bZIP, MYB, WRKY). Altogether, it results in expression of defense related genes like Pox, Pal, Lac, Chit, Glc, Lpo leading to synthesis of PHY and PHO type of antimicrobial compounds that responds to *M. oryzae* growth on the cell wall. Abbreviations: GTP, Guanosine-5’-triphosphate; UDP, Uridine diphosphate; UTP, Uridine-5’-triphosphate; PPi, Pyrophosphate; NADP^+^, Nicotinamide adenine dinucleotide phosphate; CO2, Carbondioxide; ROS, reactive oxygen species; HR, hypersensitive response; PCD, programmed cell death; TF, transcription factor; ET, ethylene; JA, jasmonic acid; PR proteins, pathogenesis-related proteins; CA, cinnamic acid; PAL, phenylalanine ammonia lyase; Glc, glucanases; Chit, chitinases; LPO, lipoxygenases; POX, peroxidases; Lac, laccases; PHO, phenolics; PHY, phytoalexin. Symbols: 1, UDP-glycosyltransferase; 2, UDP-glucose 6-dehydrogenase.

To explain the panicle blast resistance mechanism in Tetep, we have proposed a model where three crucial pathways have been depicted which culminate in strong defense response against panicle blast disease (Figure 6). The stimulus of pathogen infection in the form of pathogen associated molecular patterns (PAMP) is perceived by plant cell receptors, chitin elicitor receptor kinase 1(CERK1) and chitin elicitor-binding protein receptor (CEBiP) complex in case of rice [55]. Later, in a series of signal transduction pathways, the systemic disease response of the plant is activated which either results in resistance like in Tetep or susceptibility as in HP2216 (Supplementary Figure S7). Downstream signal transduction of pathogen infection requires BAK1, a central regulator of plant immunity [56] which was found to be highly up-regulated in Tetep. One of the major pathways responsible for fungal resistance in Tetep was found to be of a cell wall precursor pathway as evident by higher up-regulation of genes of this pathway as compared to HP2216. This pathway ultimately activates redox reaction in the plant cell wall. Conversion of Uridine Diphosphate (UDP-D) glucose to sucrose is enhanced by nearly identical to sucrose synthase 6 enzyme that is up-regulated at 48 hpi in Tetep. The early (48 hpi) up-regulation of UDP-glucose-6-dehydrogenase leads to conversion UDP-D glucose into UDP-D glucuronic acid and early generation of NA(P)D^+^ from NAD(P)H. This oxidation of nicotinamide adenine dinucleotide phosphate (NADP^+^) leads to ROS production in the plant cell wall. The ROS act as vital signaling molecules to trigger hypersensitive response in resistant hosts [57] and therefore, it is important for plants to generate ROS as soon as pathogen attacks. Here, it is observed that the genes for ROS production were immediately expressed in Tetep, but it was quite delayed in susceptible HP2216. At the same time, genes regulating electron transport, oxidative stress and peroxidase synthesis were activated and these molecules accumulated. The downstream signaling molecules perceive ROS and activate cell wall dependent kinases, receptor kinases, calcium sensor proteins, G proteins and hormonal signaling associated with jasmonic acid and ethylene in the cell. The jasmonic acid biosynthesis is also activated by another pathway where linoleic acid which is a key component of the membrane in chloroplast, and thylakoids gets released and acts as a stress signal. The activated kinases then induce the expression of various transcription factors like WRKY, MYB, ERF, bZIP which in turn activates PR and other defense responsive genes like chitinases, glucanases, PAL, Lac, Lpo, etc. Enhanced accumulation of JA and ET in the cell further activates the production of enzymes responsible for lipid metabolism and fatty acid biosynthesis. Further, activation of shikimate, arylmonoamine and phenylpropanoid pathways results in synthesis of several important secondary metabolites in plant cells (Supplementary Figure S8). Accumulation of these phenolics and phytoalexins, lignins, cutins, etc. ends up in cell wall reinforcement and also programmed cell death against fungal infection in particular or other biotic stresses in general [15,58,59].

Thus, along with basal resistance mechanisms like early defense response through ROS production and detoxification, MAPK and LRR signaling, accumulation of antimicrobial compounds and secondary metabolites, we propose cell wall modification and strengthening as major strategies of defense response in Tetep cultivar of rice. The modified cell wall may act as a barrier not allowing appressorium of pathogen to penetrate the cell at early stage and thus, finally make this cultivar resistant to panicle blast.

Besides the understanding of disease resistance mechanism, the present study also identified some of the novel genes that could play a vital role in panicle blast resistance in Tetep. For example, two genes with Von Willebrand factor type A (VWA) domain were found to be highly up-regulated in Tetep as compared to HP2216 during infection. Recently, these two genes have been identified in a panicle blast resistance Pb-bd1 locus by fine mapping [7]. This indicates that these VWA domain containing genes could possibly play an important role in blast resistance. One of these genes was also found to be up regulated in gall midge resistant rice line after gall midge attack [60]. Similarly, eukaryotic aspartyl protease domain containing protein, plasma membrane cation-binding protein 1 (PCaP1) and PMR5 family genes showed up-regulation at all the stages of infection in Tetep. *Arabidopsis* eukaryotic aspartyl protease APCB1, is involved in autophagy and fungal resistance in plants by cleaving Bcl-2-associated athanogene 6 (BAG6) protein [61]. Induction of this gene during panicle blast in our study suggests its similar role in blast resistance in rice. Up-regulation of *PCaP1* gene during *Magnaporthe* infection in Tetep, shows regulatory networks of biotic and abiotic stress may have significant cross linking, as its protein was reported to be increased earlier under salt stress in rice [62]. Another interesting gene AIR12 which codes for a DOMON domain containing protein was up-regulated in Tetep but was down-regulated in HP2216. Armadillo/β-catenin repeat family protein also showed significant up-regulation in Tetep but drastic down-regulation in HP2216. A cupin domain containing protein known as *OsGLP8*-12 which belongs to a germin-like protein family was up-regulated at 72 hpi and 96 hpi in Tetep but it was not expressed in HP2216. This gene along with its other family members constitutes a QTL that confers a broad-spectrum resistance to rice blast disease [63]. Thus, in addition to resistance governed by R genes in Tetep, these genes may account for panicle blast resistance and necessitate further experimental validation.

## 5. Conclusions

Comparative transcriptome analysis of blast resistant Tetep and susceptible HP2216 revealed the extensive impact of panicle blast disease on global gene expression patterns in both the cultivars of rice. Panicle blast resistance in Tetep is characterized by immediate up-regulation of a large number of genes involved in ROS production and detoxification, signal transduction, transcription regulation, primary defense response, hormonal regulation, secondary metabolite synthesis, and cell wall synthesis and modifications. Most importantly, several genes associated with cell wall precursor synthesis, lignin synthesis and chitinases which strengthen the cell wall and ultimately restrict the entry and spread of fungi, were predominant in Tetep compared to HP2216. Thus, our results showed that a plethora of genes and pathways activated for panicle blast resistance specifically in resistant cultivar Tetep and can be utilized in rice blast breeding program.

## Data Availability

The dataset(s) supporting the conclusions of this article are available in the Sequence Read Archive (SRA): Series -GSE136672; BioProject-PRJNA563035; SRA-SRP219797 and also as additional files in the article.

## Supplementary Materials

The following are available online at https://www.mdpi.com/2073-4425/12/2/301/s1, Figure S1: Chromosome map of differentially expressed loci in Tetep, Figure S2: Chromosome map of differentially expressed loci in HP2216, Figure S3: GO annotations of differentially expressed loci in different functional groups at all three-time intervals in HP2216 and Tetep, Figure S4: Comparative graphical presentation of different functional groups at all three-time intervals in HP2216 and Tetep, Figure S5: MAPK signaling pathway in plants, Figure S6: Metabolism overview of significant differentially expressed genes, Figure S7: Plant pathogen interaction pathway, Figure S8: Phenylpropanoid biosynthesis pathway, Table S1: Number of panicles scored according to their size of lesion of Panicle Blast according to the method proposed by Ou (Ou, S.H., 1965), Table S2: Mapping of RNA-Seq Reads Obtained from Tetep and HP2216 against Oryza sativa Nipponbare (MSU release 7)., Table S3: Details of primers used for quantitative real time PCR validation, Table S4: Significant differentially expressed loci at different time points of infection.
