# Supplementary material for: Understanding Rice-Magnaporthe Oryzae Interaction in Resistant and Susceptible Cultivars of Rice under Panicle Blast Infection Using a Time-Course Transcriptome Analysis
Source: Genes (Basel). 2021 Feb 20;12(2):301. doi: 10.3390/genes12020301 (PMC7924189; doi:10.3390/genes12020301)
Supplement: Supplementary file 1 [file genes-12-00301-s001.zip › Supplementary Figures S1-S8.pdf]

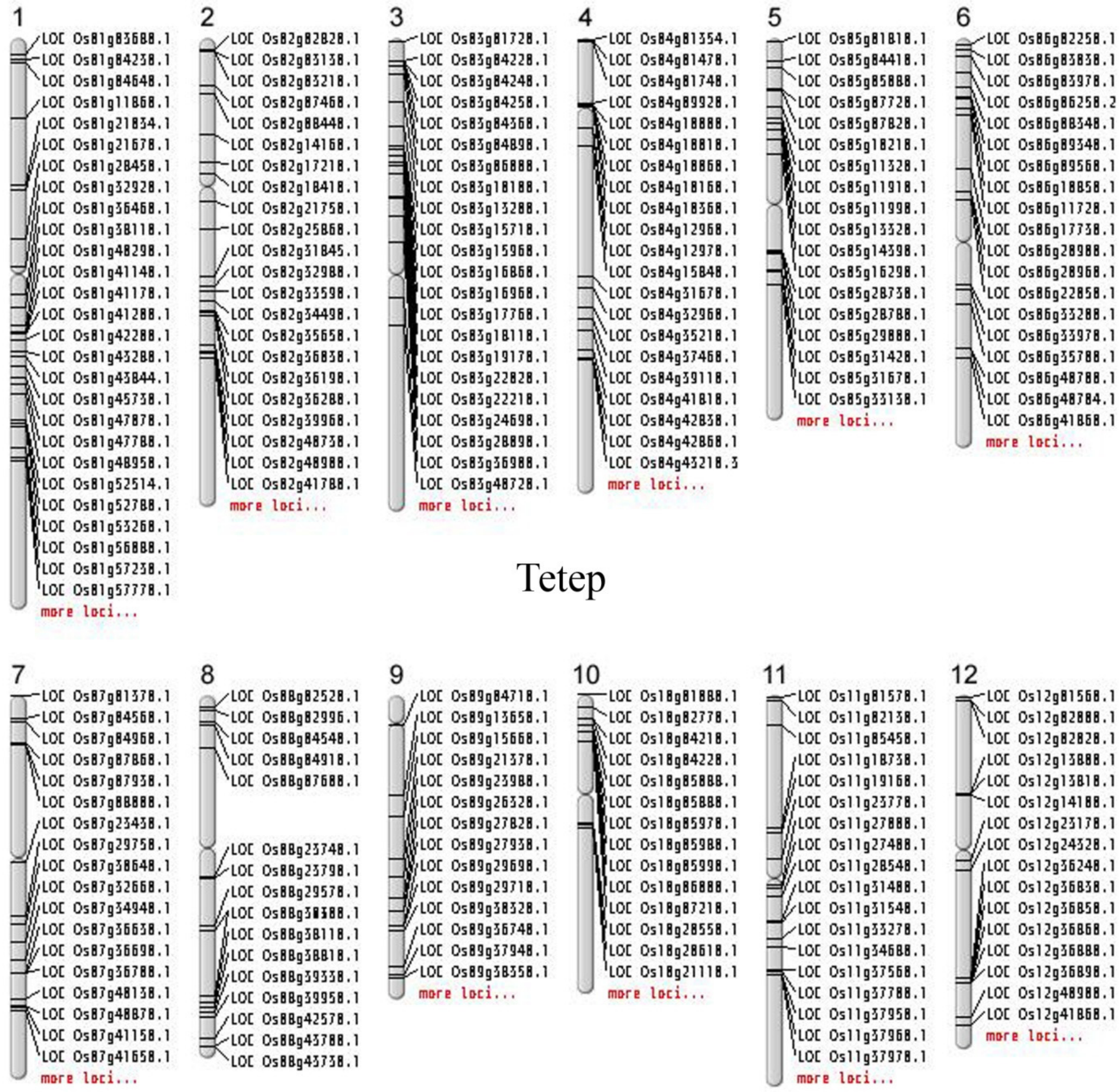

Figure S1. Chromosome map of differentially expressed loci in Tetep.

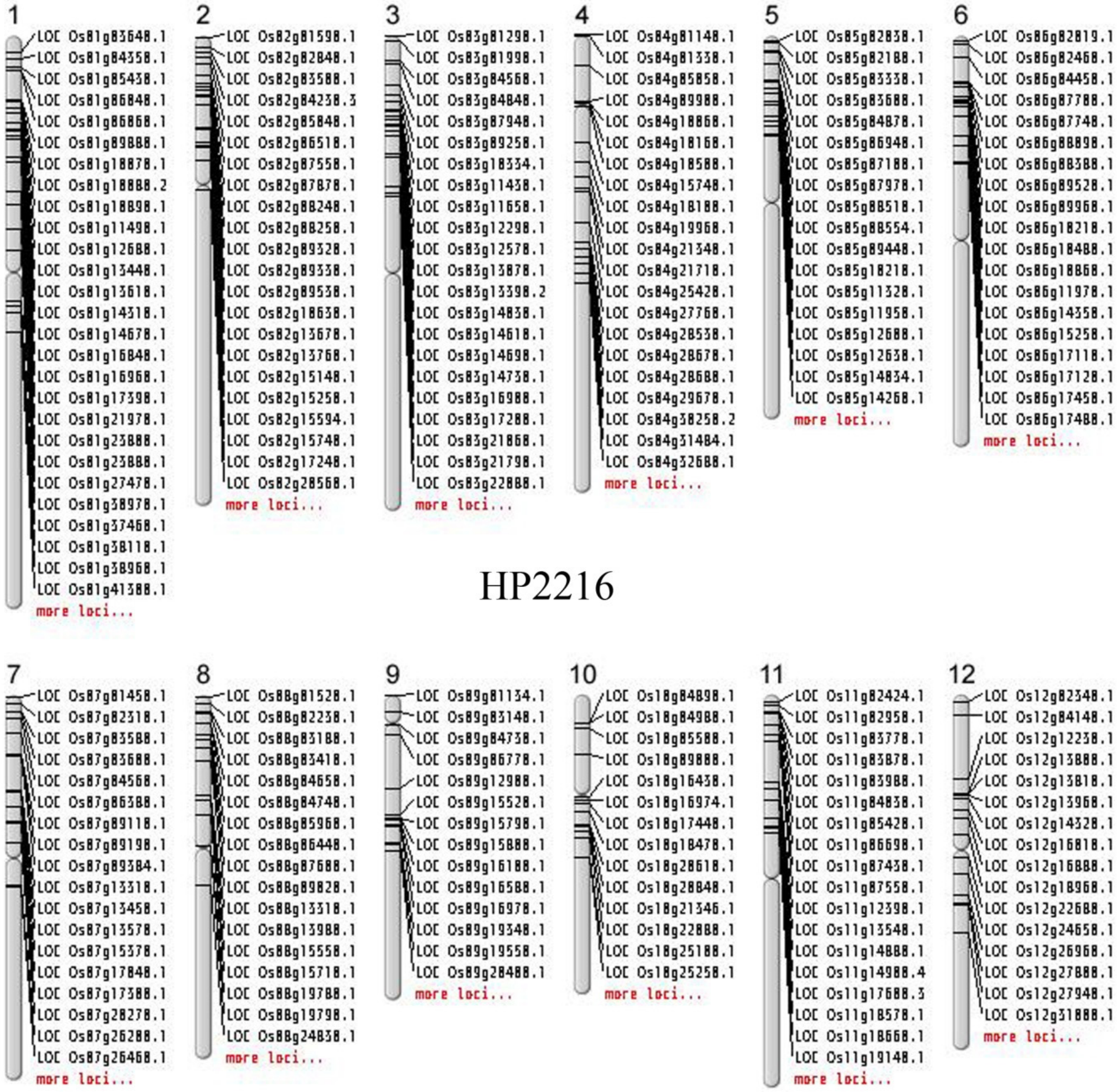

Figure S2. Chromosome map of differentially expressed loci in HP2216.

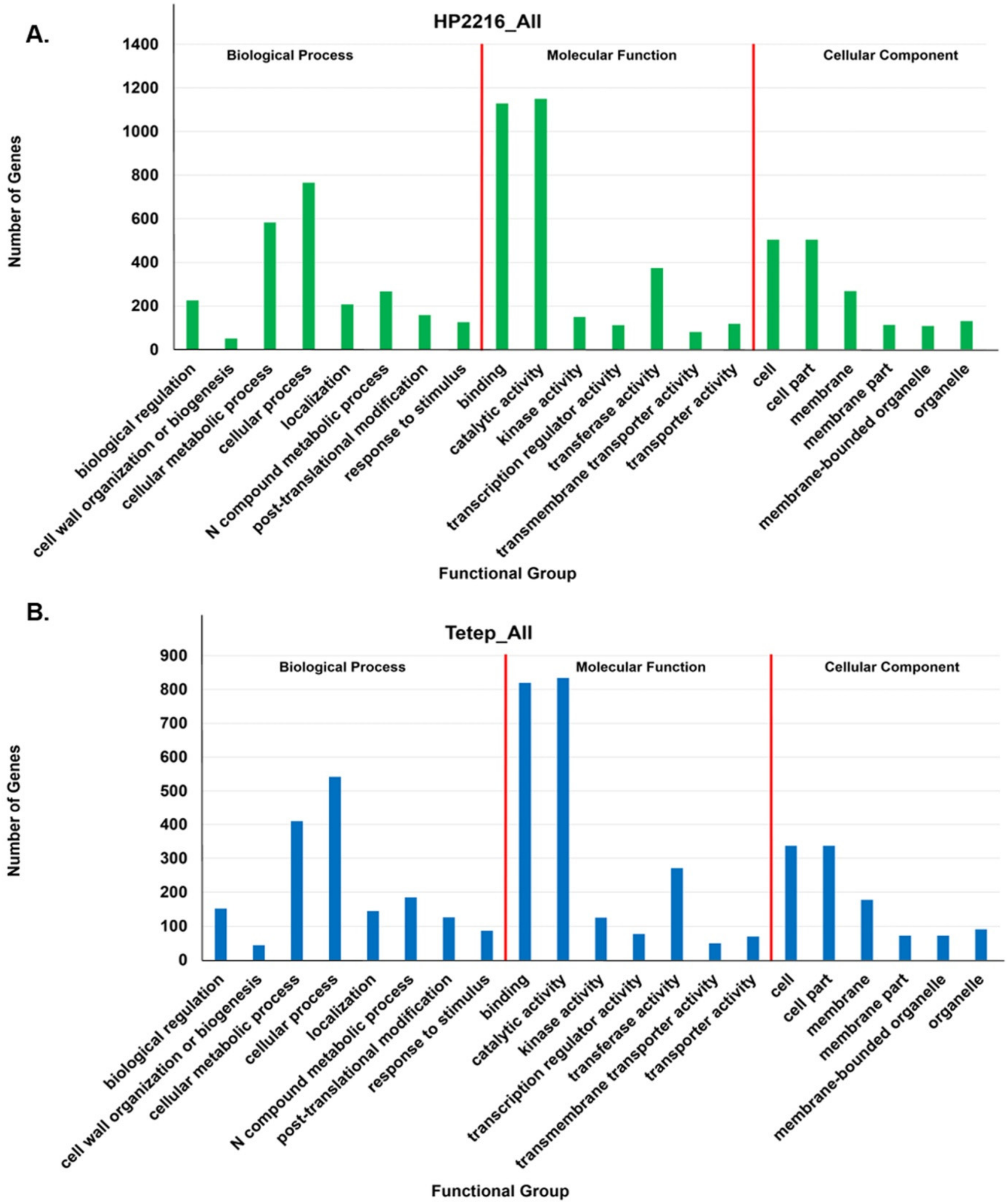

Figure S3. GO annotations of differentially expressed loci in different functional groups at all three time intervals in HP2216 and Tetep.

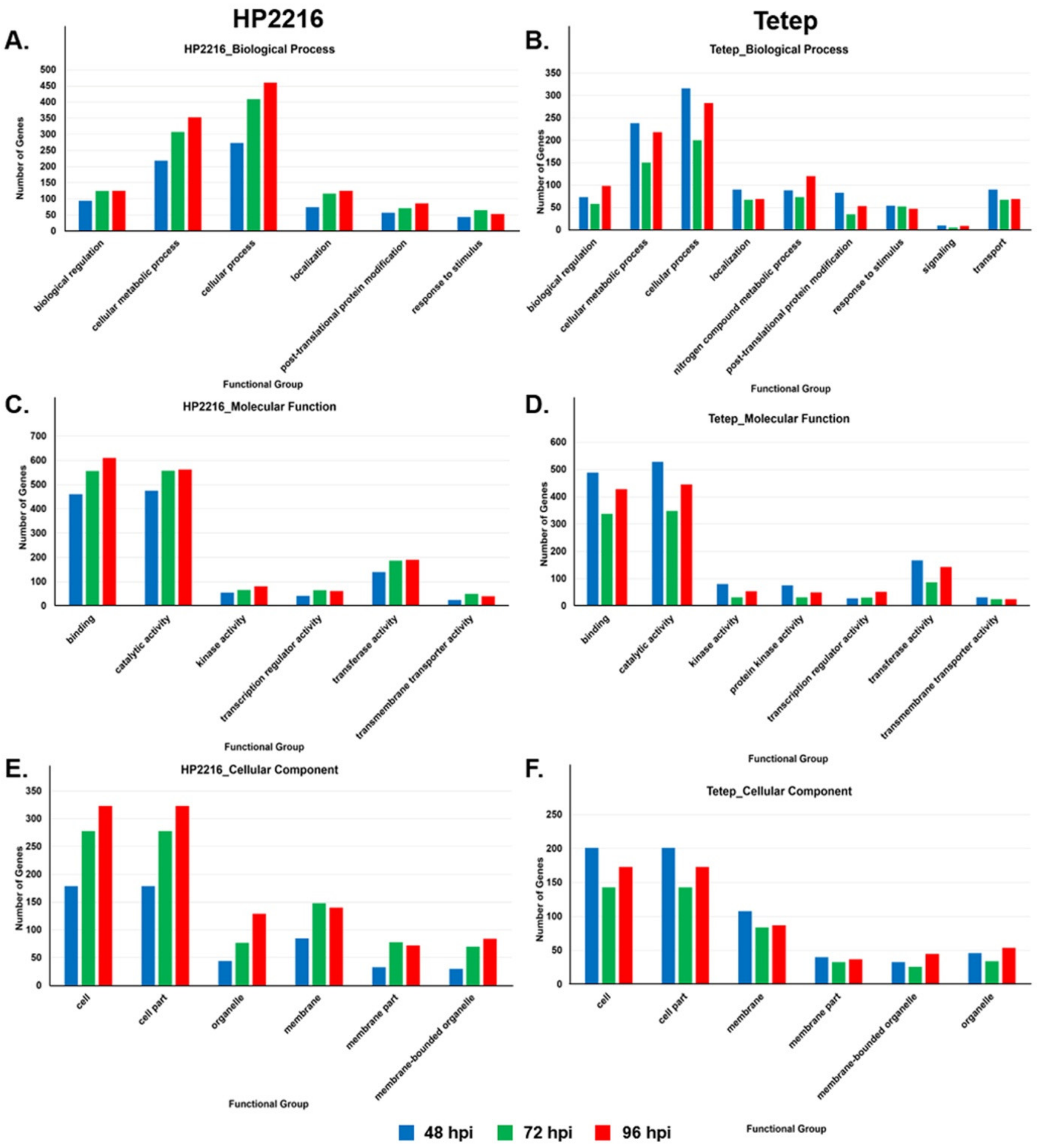

Figure S4. Comparative graphical presentation of different functional groups at all three time intervals in HP2216 and Tetep.

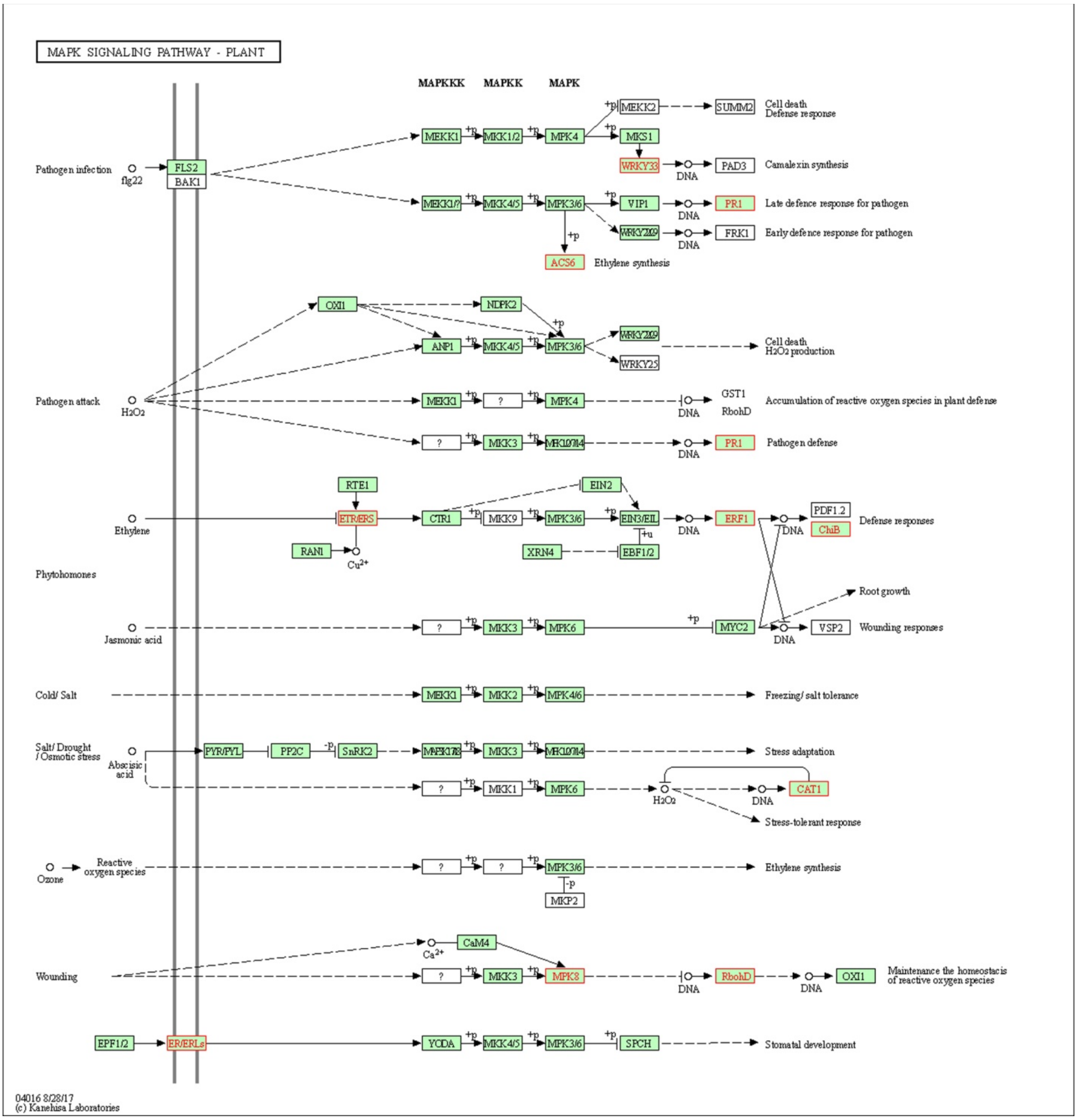

Figure S5. MAPK signaling pathway in plants.

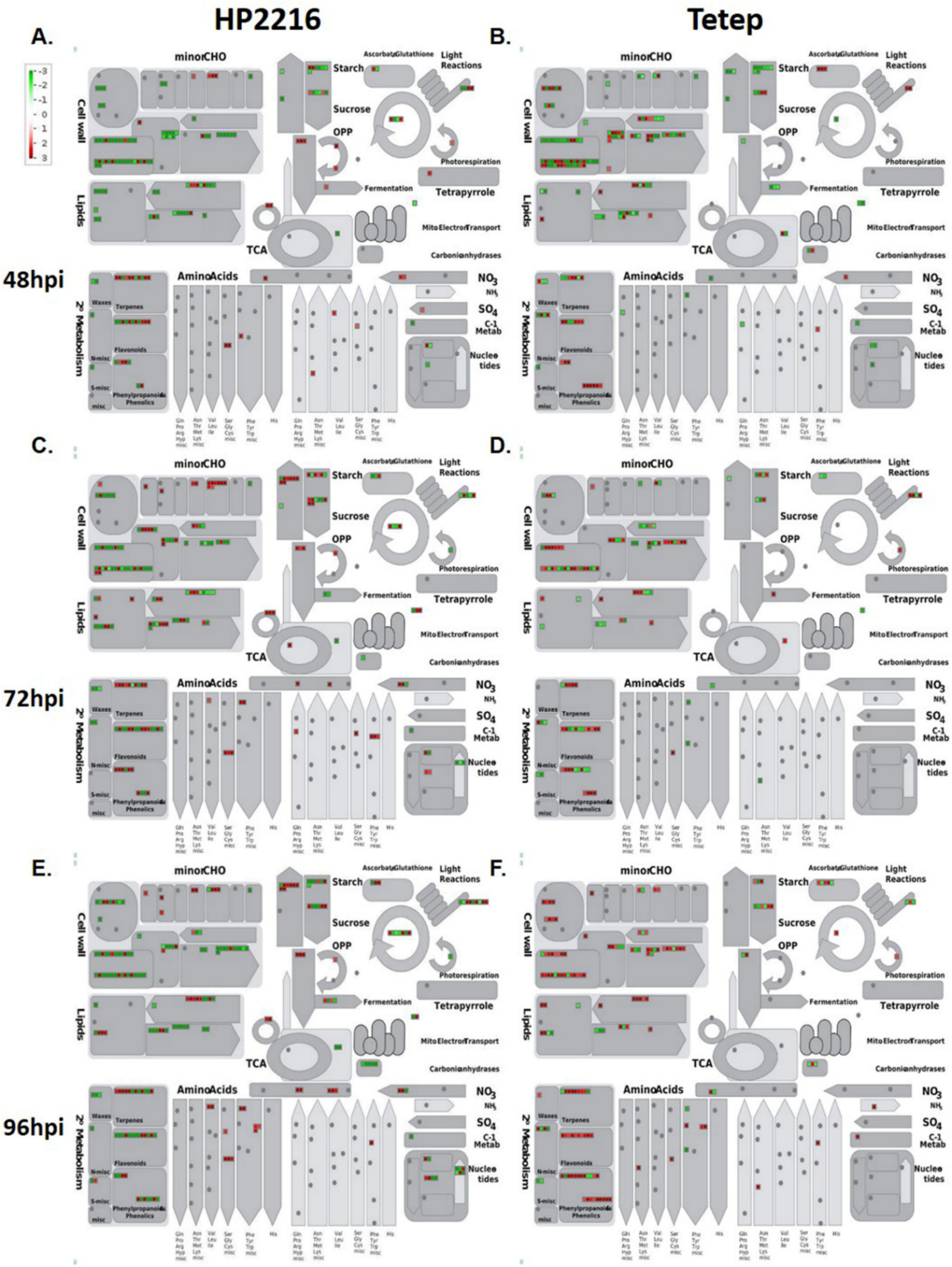

Figure S6. Metabolism overview of significant differentially expressed genes.

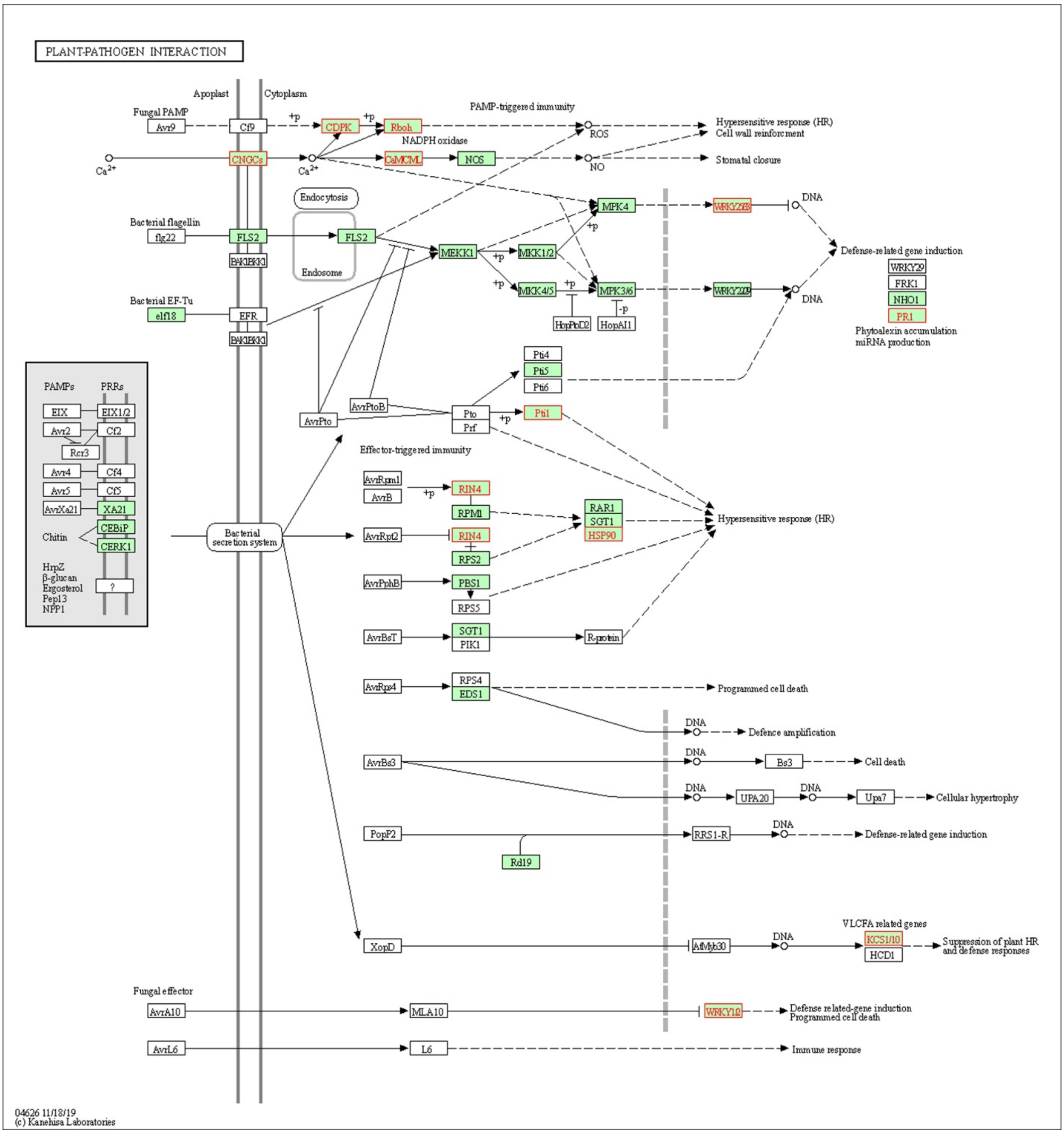

Figure S7. Plant pathogen interaction pathway.

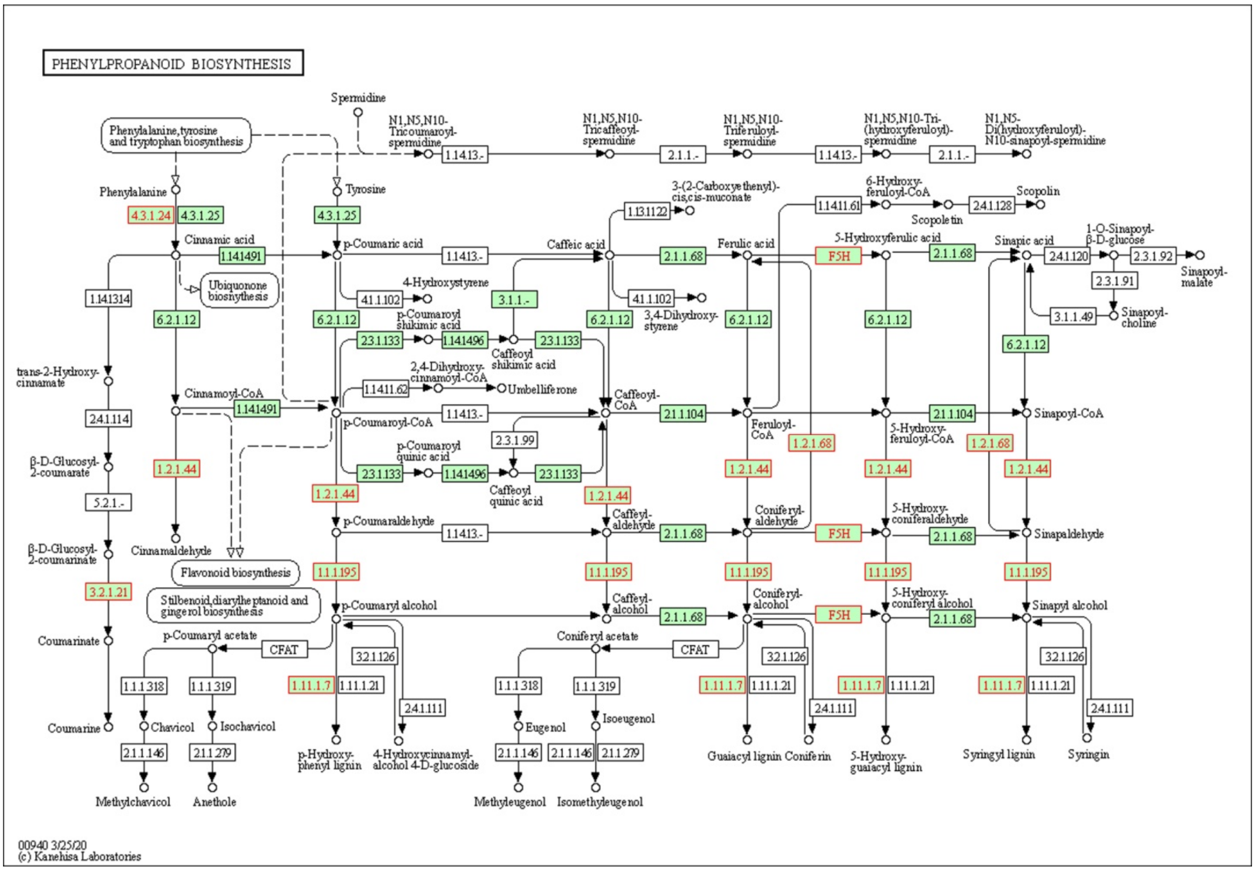

Figure S8. Phenylpropanoid biosynthesis pathway.
