# Supplementary material for: Understanding Rice-Magnaporthe Oryzae Interaction in Resistant and Susceptible Cultivars of Rice under Panicle Blast Infection Using a Time-Course Transcriptome Analysis
Source: Genes (Basel). 2021 Feb 20;12(2):301. doi: 10.3390/genes12020301 (PMC7924189; doi:10.3390/genes12020301)
Supplement: Supplementary file 1 [file genes-12-00301-s001.zip › supplementary Tables S1-S3.pdf]

Table S1. Number of panicles scored according to their size of lesion of Panicle Blast according to the method proposed by Ou (Ou, S.H., 1965). "0" is for without any evidence of infection; "1" for a lesion smaller than 0.5 mm of diameter with no sporulation; "2" is for the lesion ranges between 0.5 to 1 mm and brown in color and without sporulation; "3" is when lesion takes a round or elliptical shape and ranges between 1-3 mm in diameter, having grey colored center and brown margins, these type of lesions are capable of sporulation; "4" is for proper necrotic grey centered with brown margin lesions, length varies from 3 mm or above, and are capable of sporulation; and "5" in this the lesion is of same as in described in 4 but with more area covered. Genotypes with scoring of lesions around 0 to 3 were considered as resistant reaction, and those with 4 to 5 were considered as susceptible reaction against the blast disease.

Scoring	HP2216				Tetep			
	Mock	48 hpi	72 hpi	96 hpi	Mock	48 hpi	72 hpi	96 hpi
**0**	25	0	0	0	25	16	15	15
**1**	0	6	0	0	0	9	10	10
**2**	0	4	2	0	0	0	0	0
**3**	0	7	5	1	0	0	0	0
**4**	0	8	6	2	0	0	0	0
**5**	0	0	12	22	0	0	0	0

Table S2. Mapping of RNA-Seq Reads Obtained from Tetep and HP2216 against Oryza sativa Nipponbare (MSU release 7). R1: Biological replicate 1, R2: Biological replicate 2; hpi: hours post infection.

Sample	Total Raw Reads	Total Preprocessed Reads (Left+Right)	Total Reads Aligned	Multi Aligned Reads (Left + Right)	Total Reads Aligned %	Total Unaligned Reads %
**Susceptible line**						
**HP2216_48 hpi R1**	22,996,818	22,815,936	19,386,788	593,518	85	3.1
**HP2216_48 hpi R2**	21,230,382	20,216,986	14,205,622	568,932	70.3	4
**HP2216_72 hpi R1**	42,404,536	42,235,521	37,181,937	4,609,385	88	12.4
**HP2216_72 hpi R2**	23,142,268	22,273,058	17,608,583	1,876,575	79.1	10.7
**HP2216_96 hpi R1**	45,546,236	45,257,792	39,341,401	4,778,432	86.9	12.1
**HP2216_96 hpi R2**	21,701,598	20,963,602	17,771,260	2,713,392	84.8	15.3
**HP2216_Mock R1**	47,109,206	46,768,736	41,225,139	957,667	88.1	2.3
**HP2216_Mock R2**	21,392,421	20,672,630	17,704,067	2,709,972	85.6	15.3
**Resistant Line**						
**Tetep_48 hpi R1**	42,034,770	41,756,080	36,732,185	670,900	88	1.8
**Tetep_48 hpi R2**	21,946,596	20,813,520	13,841,691	259,150	66.5	1.9
**Tetep_72 hpi R1**	42,194,552	41,880,774	36,958,832	673,142	88.2	1.8
**Tetep_72 hpi R2**	22,017,790	20,952,144	15,876,148	312,849	75.8	2
**Tetep_96 hpi R1**	46,289,178	45,940,760	40,544,425	755,308	88.3	1.9
**Tetep_96 hpi R2**	22,198,855	21,537,207	18,169,151	332,915	84.4	1.8
**Tetep_Mock R1**	44,181,464	43,800,508	39,189,039	930,869	89.5	2.4
**Tetep_Mock R2**	23,327,572	22,570,334	19,217,509	421,213	85.1	2.2
**Average**	31,857,140	31,278,474	26,559,611	1447,764	83.4	5.7
**In million**	31.857140	31.278474	26.559611	1.447764	83.35	5.69

Table S3. Details of primers used for quantitative real time PCR validation**.**

Locus_id	Annotation	Forward Primer	Reverse Primers
**LOC_Os01g62480**	laccase precursor protein, putative, expressed	GAACAACACCAACGTGAA-GAC	CTTGGCCGATCACGAAGAA
**LOC_Os02g43790**	ethylene-responsive transcription factor, putative, expressed	CCGAGGACATGGTCGTGTA	AGGAAGCTGCCGATGGA
**LOC_Os03g16960**	cysteine-rich repeat secretory protein 55 precursor, putative, expressed	CAACTAC-CAATCCGACTCAAGA	CTGCACGTTCACAAGAATCAC
**LOC_Os03g17200**	plant-specific domain TIGR01589 family protein, expressed	GTGCAGCACCTGATAGA-GAAA	TCTCCAGCTCCTTCCATACT
**LOC_Os03g46860**	helix-loop-helix DNA-binding protein, putative, expressed	AGGATCCACGGCGAGAATAA	TGACATTGGTGTGCGTGATG
**LOC_Os04g56430**	cysteine-rich receptor-like protein kinase, putative, expressed	GCACTTTGCTCGTTCTCCT	CAGGTTCAGGAGGTTGTTCTC
**LOC_Os04g59150**	peroxidase precursor, putative, expressed	GCATCATTCTTGCGCTCTTG	CGTAGTGATTGGAAGATGG-TAGG
**LOC_Os05g07820**	leucine-rich repeat receptor protein kinase EXS precursor, putative, expressed	GATCAGCTCCTCCATT-GTTCTT	GTGGCAGATGTTGTGTCTACT
**LOC_Os06g08360**	ethylene-responsive element-binding protein, putative, expressed	GCAACTGCAACCACTTCTG	GGATGACGCAGGTGAAGA
**LOC_Os06g09860**	expressed protein	CGCTCAAGGTGTTCGACAT	ACGTAGCAGAGGTACTGGTT
**LOC_Os06g12210**	helix-loop-helix DNA-binding domain containing protein, expressed	GATCAAC-GAGCTCATCTCCAA	GGTGCAGGCTCTTGATGTA
**LOC_Os08g43700**	OsSAUR36 - Auxin-responsive SAUR gene family member, ex-pressed	CGGTGATGGAG-TATGTCATGTG	CATCACAATGGAACTGAGGA-GAG
